# Nanoplasmonic fiber tip probe detects significant reduction of intracellular Alzheimer’s disease-related oligomers by curcumin

**DOI:** 10.1038/s41598-017-05619-z

**Published:** 2017-07-18

**Authors:** Feng Liang, Yu Wan, Diane Schaak, Joseph Ward, Xunuo Shen, Rudolph E. Tanzi, Can Zhang, Qimin Quan

**Affiliations:** 1 Rowland Institute at Harvard University, Cambridge, MA 02142 United States; 2 Genetics and Aging Research Unit, MassGeneral Institute for Neurodegenerative Disease, Department of Neurology, Massachusetts General Hospital and Harvard Medical School, Charlestown, MA 02129 United States; 3 Department of Neurology, Qingdao Municipal Hospital, Qingdao University, Qingdao, P. R. China

## Abstract

Considerable evidence shows critical roles of intracellular pathogenic events of Alzheimer’s disease (AD). In particular, intracellular amyloid-β accumulation and oligomerization are early AD pathologic processes, which may lead to changes in inflammatory molecules and other AD-related pathological components. Curcumin and its analogs have been identified as potential drug candidates for AD. However, the effects of curcumin on intracellular AD pathologic processes remain largely unknown. Here we utilized a recently developed nanoplasmonic fiber tip probe (nFTP) technology and investigated whether curcumin leads to intracellular AD pathologic changes. We showed that our nFTP technology could robustly detect intracellular AD-related protein changes caused by a well-known inflammation inducer and a familial AD mutation. Intriguingly, curcumin remarkably reduced the level of intracellular oligomers while modestly reduced the level of an inflammatory cytokine. Thus, our results provided evidence that curcumin’s mechanism of action in attenuating AD pathology is through a major role of decreasing oligomerization.

## Introduction

Alzheimer’s disease (AD) is a devastating neurodegenerative disorder and the primary cause of dementia in the elderly^1^. Presently there are no effective therapeutics available to modify disease progression. Although the underlying mechanisms of AD have not been completely elucidated, it is characterized by two pathological hallmarks: β-amyloid plaques primarily comprised of a small protein - amyloid-β (Aβ) and neurofibrillary tangles composed of phosphorylated tau proteins^2–4^. Emerging evidence showed that immunity and inflammatory events are closely related to these two pathologic hallmarks^5–7^.

Strong evidence supports a use for curcumin and analogs in development of AD therapeutics. Curcumin (diferuloylmethane) is the active constituent and yellow pigment of turmeric, widely used in Indian culinary preparations, which has been suggested to be coincident with one of the lowest prevalence rates of AD in India^8, 9^. Low bioavailability and limited blood-brain-barrier penetration decrease the effects of curcumin in clinical effectiveness. Thus, curcumin analogs with stronger effects and higher bioavailability are being developed as potential AD therapeutics^10–12^. Curcumin interferes with AD pathology via several mechanisms, including anti-inflammatory, anti-phosphorylation and anti-oligomerization effects. Studies in AD mouse model suggest that curcumin suppresses inflammatory cytokines and decreases the level of Aβ proteins^13^. Clinical studies also suggest that curcuminoids, derivatives of curcumin, enhance the macrophage uptake^14^ and improve patients’ AD clinical symptoms^15, 16^. Additionally, curcumin markedly decreases conditioned medium Aβ levels in cell-based studies^17^ and decreases existing β-amyloid plaques in AD mouse models^18, 19^. Binding between curcumin and Aβ oligomers has also been identified in cell-free studies^20^.

While these AD model-based studies confirm strong effects of curcumin in decreasing AD pathology, the roles of curcumin on intracellular AD pathologic events remain largely unknown. Numerous studies showed that Aβ can generated and accumulate intracellularly and affect AD pathology^21–33^. Aβ can also transfer intercellularly, thus playing key roles in AD progression^34, 35^. Notably, intracellular protein aggregation and oligomer accumulation are early events in development of AD pathology^23, 36, 37^. Presently, we focus on investigating the effects of curcumin on AD intracellular events through our recently developed nanoplasmonic fiber tip probe (nFTP) technology^38, 39^. Particularly, this technology provides a quantitative approach to robustly measure intracellular proteins in live, single cells.

Here we utilized a well-characterized AD cell model, the Chinese hamster ovary (CHO) cells stably over-expressing familial AD (FAD) “*Indiana*” mutation in APP (also known as 7PA2) with high expression levels of Aβ and oligomers^23, 40^. Furthermore, we asked whether a well-known pharmacological inflammation inducer (lipopolysaccharide; LPS) may influence AD intracellular events that can be attenuated by curcumin. We showed that both genetic and pharmacological components led to pronounced changes in intracellular AD-related proteins, including Aβ42, A11-reactive oligomers and an inflammatory cytokine - tumor necrosis factor-α (TNF-α). Interestingly, we showed that curcumin displayed remarkable reductions in the levels of intracellular oligomers, significant but less pronounced reductions in Aβ42, and the least pronounced change in TNF-α. Collectively, we provided compelling evidence for the first time that a strong mechanism of action in curcumin toward therapeutics in AD may be through reducing intracellular AD-related proteins, particularly via decreasing oligomerization.

## Results and Discussion

### nFTP detects AD pathologic events induced by LPS or an FAD mutation

**Figure 1:**
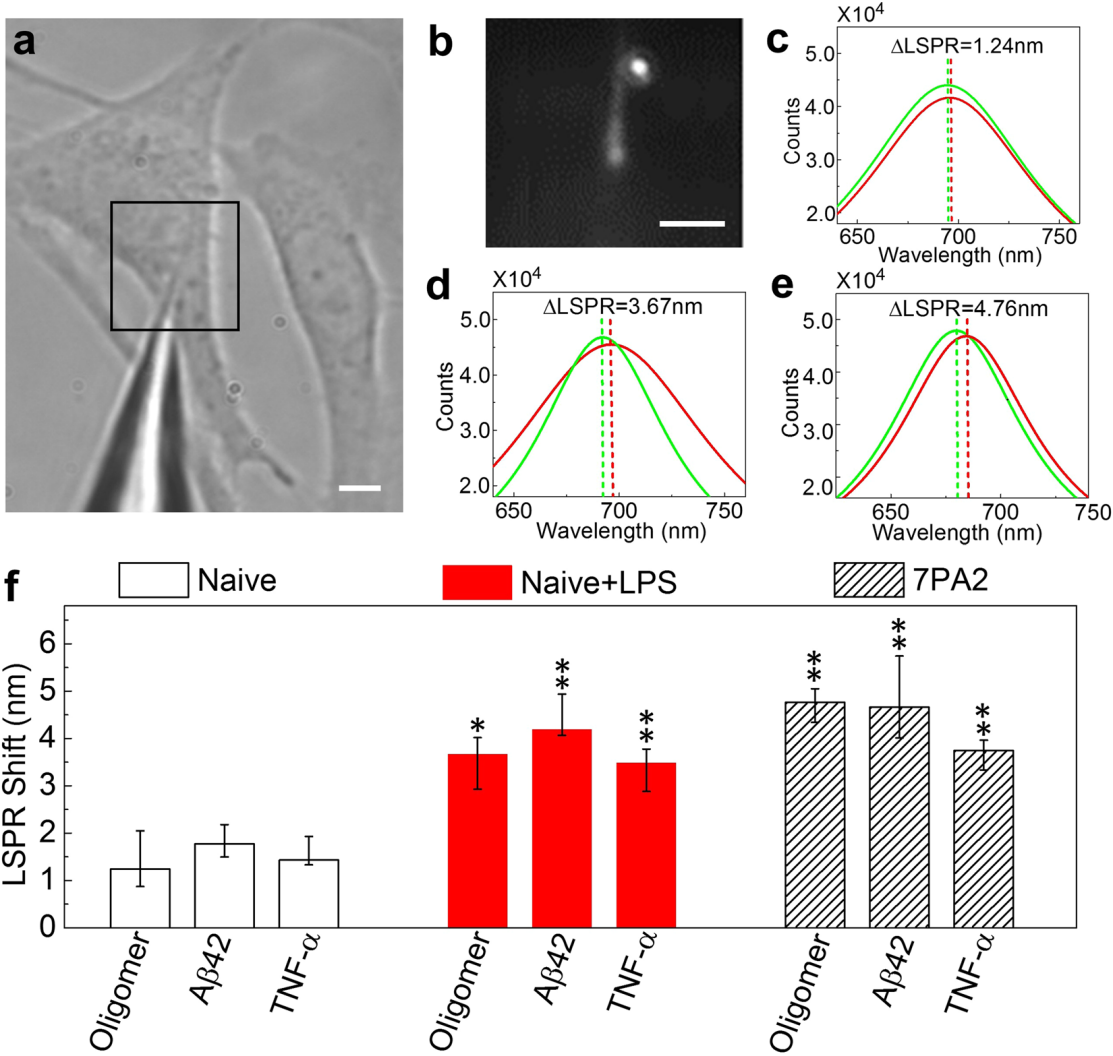
Nanoplasmonic fiber tip probe (nFTP) measures intracellular pathological proteins related to Alzheimer’s disease (AD). (**a**) The optical image of the nFTP probe and a target live Chinese hamster ovary (CHO) cell. Scale bar, 5 μm. (**b**) Zoom-in image of the tip area (box in **a**) using an electron multiplying charge coupled device (EMCCD), where a bright white dot shows the strong localized surface plasmon resonance (LSPR) signal from the gold nanorod. Scale bar, 5 μm. (**c**) A11-related LSPR signal from a CHO-naïve cell. The green and red curves correspond to LSPR spectra measured before inserting nFTP into the cell and after retracting nFTP from the cell, respectively. (**d**) A11-related LSPR shift for a CHO-naïve cell incubated in 10 ng/mL LPS overnight (3.67 nm), displaying a significant increase from control (without LPS treatment, 1.24 nm in **c**). (**e**) A11-related LSPR shift is remarkably higher (4.76 nm) for CHO cells stably over-expressing familial AD (FAD) “Indiana” mutation in APP (also known as 7PA2). (**f**) Intracellular A11-reactive oligomers, Aβ42 and TNF-α proteins in CHO naïve, LPS induced CHO-naïve and 7PA2 cells. Error bars indicate the mean, minimum and maximum LSPR shift obtained from probing different single cells (N ≥ 3). The *p* values are obtained from the t-test analysis between the LPS and CHO-naïve group, and between 7PA2 cell and CHO-naïve group. *p* ≤ *0.05* (*), *p* ≤ *0.01* (**) and *p* ≤ *0.001* (***).

Our nFTP system is comprised of a nanoscale optical fiber with a sub-100 nm tip (Fig. 1a), where a single gold nanorod biosensor is attached to its end (Fig. 1b). The free electrons on the gold surface can be collectively excited by visible photons directly from the optical fiber. The geometry of the gold nanorod tunes the collective oscillation to a resonance spectrum centered at 700 nm (Fig. 1c), so called localized surface plasmon resonance (LSPR). We functionalized and optimized the gold nanorod surface with various antibodies in this study. Thus, LSPR spectrum will display a specific resonance shift when its target antigens bind on the gold surface. Because of the sub-100 nm geometry of the tip, the fiber tip probe can insert into a single cell without affecting its viability and furthermore measures the intracellular protein concentrations. The sensitivity of the nFTP ranges from 0.5 nM to 0.5 μM for tested proteins which have affinity values at 10–100 nM. nFTP technology has several unique advantages compared to fluorescent imaging or immunoassays. First, it offers quantitative measurement of intracellular soluble proteins with higher sensitivity than immunoblotting. Second, nFTP measures intracellular protein levels in individual cells, therefore, information on both averaged value and deviations due to cell heterogeneity is preserved. Third, being a live-cell approach, nFTP can track the protein levels in the same cell over a time course.

Notably our current nFTP is designed to measure soluble proteins that diffuse across the cell which lead to an averaged level in cytoplasm. Our measurement procedure takes the following steps. First, the fiber tip was micro-controlled to the vicinity of a target cell, where a baseline LSPR spectrum was taken. Next, the tip was inserted into the cytoplasm and was incubated for 2 min. Note that the time scale for a protein (~30 kDa) to diffuse across the cell is on the order of 10 seconds. After incubation, the tip was retracted from the cell and another LSPR spectrum was measured. The baseline and final resonance spectra were then fitted using Lorentzian functions, by which the central peak positions were obtained. The shift of the central peak position before and after incubation indicates the protein level inside the cell (Fig. 1c–e). 1000 LSPR spectra were taken and averaged in each measurement.

Next, we utilized our nFTP system and characterized various AD-related proteins (A11-oligomers, Aβ42 and TNF-α) in CHO-naïve cells at the baseline and those treated with lipolysaccharides (LPS). LPS is a well-known immune response inducer which elevates levels of Aβ42^41^. We used previously reported antibodies, including the anti-oligomer A11 (Millipore AB9234; polyclonal), anti-β amyloid 1-42 (reactive to Aβ42, but not Aβ40 or full-length APP, Abcam Ab10148; polyclonal) and anti-TNF-α (Abcam Ab9739; polyclonal), respectively. We showed that LSPR from nFTP functionalized with A11 displayed a resonance shift 1.24 nm in naive cells (Fig. 1c). Notably, our results suggested that these A11-reactive oligomers displayed high solubility, consistent with previous findings^36, 42^. Treatment of 10 ng/mL LPS overnight led to an increase in A11-related LSPR to 3.67 nm (Fig. 1d). In addition, LSPR shifts for Aβ42 and TNF-α in cells treated with LPS were significantly higher than the cells treated with control (*p = 0.0016* for Aβ42 and *p = 0.0049* for TNF-α, see Fig. 1f). Thus, our nFTP could robustly detect the changes of AD intracellular events induced by LPS. Next, we studied the intracellular AD-related events in APP^Indiana^ mutation containing 7PA2 cells. We showed that the genetic mutation led to a robust increase in A11-related LSPR to 4.76 nm (Fig. 1e). Furthermore, LSPRs for Aβ42 and TNF-α in 7PA2 cells were also significantly increased compared to naive cells (*p = 0.0053* for Aβ42 and *p = 0.0013* for TNF-α, see Fig. 1f). Our results suggest that the changes in Aβ peptides from FAD mutations acts as driving force for immune responses, consistent with the suggested roles of inflammation in AD^5^.

### nFTP analysis on curcumin’s effect on AD pathogenic proteins

**Figure 2:**
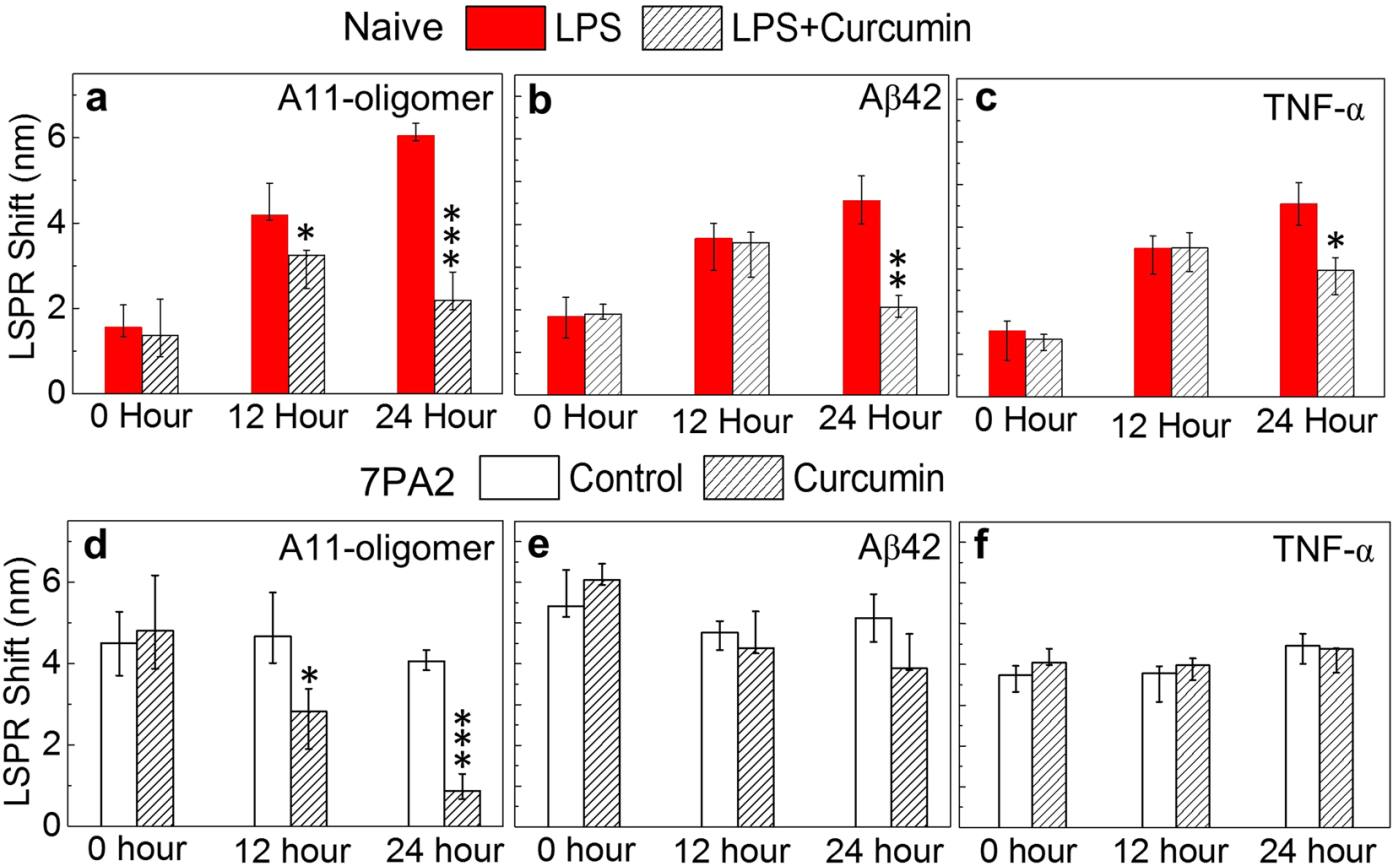
Curcumin’s effect on AD pathologic events caused by LPS or a FAD mutation. (**a**–**c**) Curcumin decreased the changes of AD-related proteins induced by LPS in CHO-naïve cells. The intracellular A11- reactive oligomers (**a**), Aβ42 (**b**), TNF-α (**c**) were measured using nFTP. The effects of curcumin on these proteins were compared between the LPS group and the LPS/curcumin co-treatment group. (**d**–**f**) 7PA2 cells stably overexpressing APP^Indiana^ were used to further evaluate curcumin’s effects on AD-related proteins. The intracellular A11-oligomers (**d**), Aβ42 (**e**) and TNF-α (**f**) were measured using nFTP technique. N ≥ 3; *p* ≤ *0.05* (*), *p* ≤ *0.01* (**) and *p* ≤ *0.001* (***).

After validating our nFTP technology for AD-related intracellular processes, we asked whether curcumin might display therapeutic effects on these events. First, we studied how curcumin changed LPS-induced AD pathologic events in CHO-naïve cells. We treated cells with vehicle [phosphate-buffered saline (PBS)], 10 ng/mL LPS alone, or 10 ng/mL LPS and 25 μM curcumin combined, for 12 and 24 hours. LPS increased levels of A11-reactive oligomers, Aβ42 and TNF-α, compared to controls (Fig. 2a–c). We found that a combination of LPS and curcumin displayed a significant effect in decreasing A11-oligomer levels compared to LPS alone (*p = 0.025*) after 12 hours. Oligomers were further remarkably reduced (*p = 0.0002*) after 24 hours (Fig. 2a). In contrast, no significant reduction in Aβ42 (Fig. 2b) or TNF-α (Fig. 2c) was observed after 12 hours. After 24 hours, reductions in Aβ42 (*p = 0.002*, Fig. 2b) and TNF-α (*p = 0.013*, Fig. 2c) were observed compared to control. Thus, these results broadened the knowledge of curcumin’s intracellular effects and showed its strong effects in attenuating an immune inducers’ actions in affecting AD-related pathologic changes.

We then evaluated the effect of curcumin in APP^Indiana^ over-expressing 7PA2 cells in a 12 and 24-hour time course. A decrease in A11-oligomers was observed after 12 hours compared to control (*p = 0.03*, Fig. 2d). No significant reductions were observed for Aβ42 or TNF-α proteins (*p* > 0.05, Fig. 2e,f). After 24 hours, A11-oligomers were further reduced remarkably (*p = 0.0002*). In comparison, no significant reductions were observed in either Aβ42 or TNF-α (*p* > 0.05, Fig. 2e,f). Collectively, these results strongly indicate a major role of curcumin in reducing oligomers inside cells, dominating its influences on Aβ42 peptide and anti-inflammation effect.

### Validation of curcumin’s effects using fluorescent imaging and immunoblot analysis

**Figure 3:**
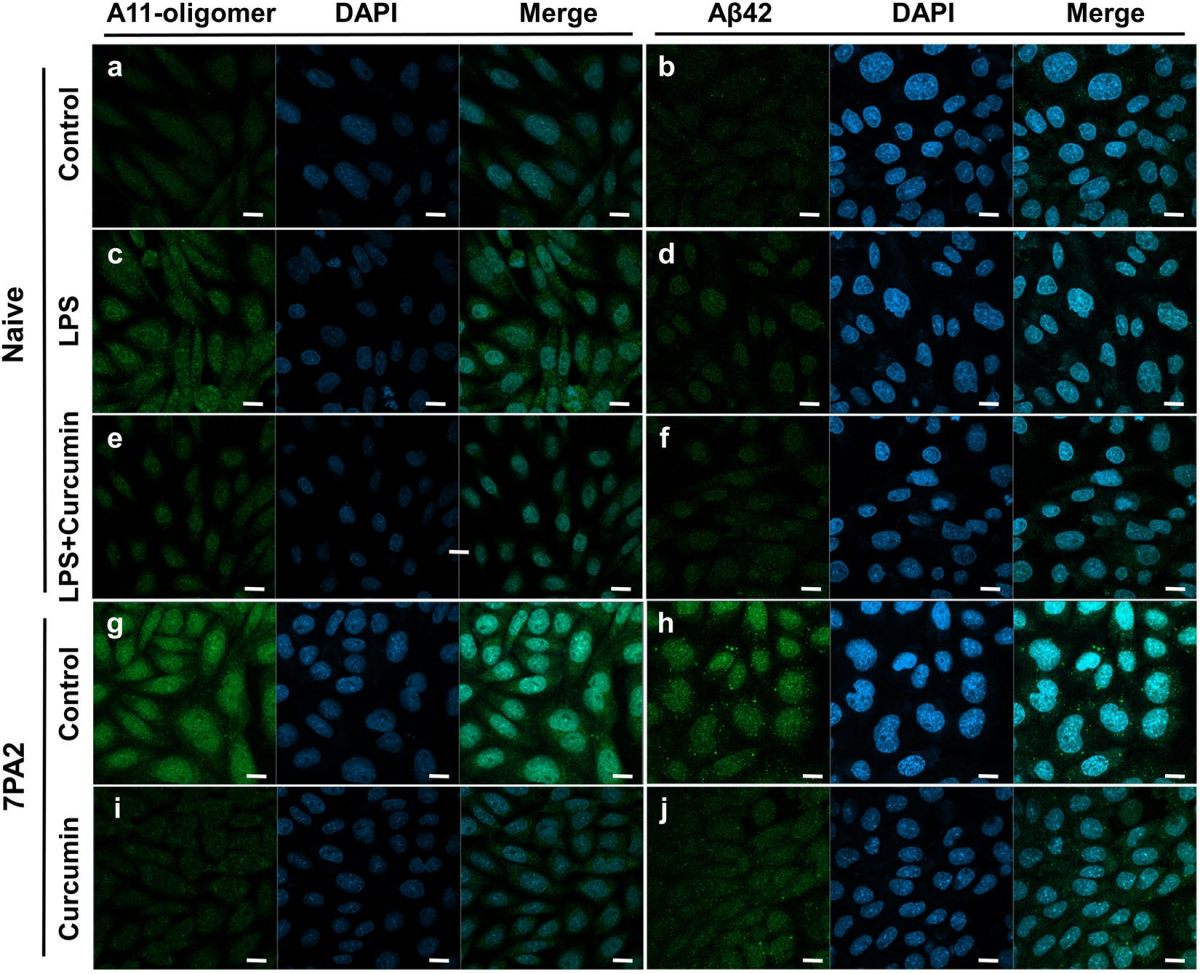
Fluorescent immunostaining analysis showing the effects of curcumin. CHO-naïve were treated with PBS (**a**,**b**), LPS (**c**,**d**) or a combination of curcumin and LPS (**e**,**f**) overnight. 7PA2 cells were treated without (**g**,**h**) or with (**i**,**j**) curcumin. The cells were then applied to fluorescent immunostaining using A11 antibody, anti-Aβ42 antibody and DAPI. (**a–d**) LPS elevated levels of A11-oligomers and Aβ42 compared to control. (**e,f**) Combined curcumin with LPS significantly reduced the level of A11-oligomers, but not Aβ42, compared to LPS alone. (**g,i**) Curcumin significantly reduced level of oligomers in 7PA2 cells compared to control. (**h,j**) Curcumin slightly reduced Aβ42 level compared to control. Scale bar, 10 μm.

Next, we used fluorescent immunostaining to evaluate curcumin’s effect to further support our nFTP results. CHO-naive cells were treated with control (PBS), LPS (10 ng/mL) or a combined treatment of LPS (10 ng/mL) and curcumin (25 μM) overnight. We showed that LPS increased both A11 antibody (Fig. 3a,c) and Aβ42 antibody-stained fluorescent signals (Fig. 3b,d) compared to control. When curcumin was co-incubated with LPS, a significant reduction in A11-stained fluorescent signals was observed (Fig. 3e) compared to LPS alone (Fig. 3c). However, no significant effect was observed for Aβ42-stained fluorescent signals (Fig. 3d,f). Moreover, in 7PA2 cells, we found a remarkable reduction in A11-related fluorescent signals (Fig. 3g,i) and a minor reduction in Aβ42-related fluorescent signals (Fig. 3h,j) after treated with 25 μM curcumin overnight. Collectively, the results of fluorescent immunostaining analysis were consistent with the nFTP measurements, confirming a major role of curcumin in reducing oligomers.

**Figure 4:**
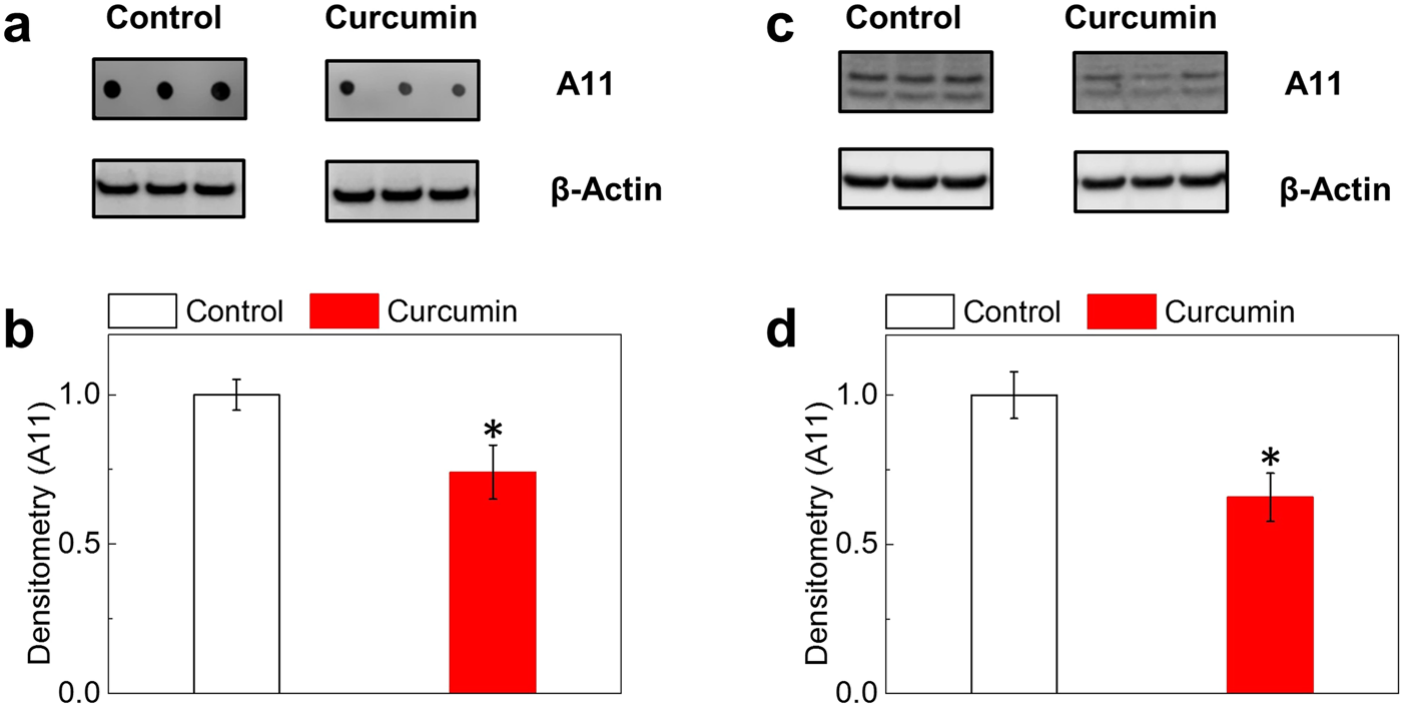
Immuno-blotting analyses of oligomers in APP^Indiana^-overexpressing 7PA2 cells. 7PA2 cells were treated with control (DMSO) or 25 μM curcumin overnight. Cells were then harvested and cell lysates were applied to immune-blotting analysis using A11 antibody. The house-keeping protein, β-actin, was used as control for normalization. (**a**,**b**) Curcumin reduced A11-reactive oligomers via dot-blotting analysis compared to control. (**c,d**) Curcumin significantly reduced A11-reactive oligomer levels via Western blotting analysis compared to control. N ≥ 3; *p* ≤ *0.05* (*).

We next further validated curcumin’s anti-oligomerization effect using immuno-blotting analysis. We treated 7PA2 cells with control (DMSO) or 25 μM curcumin overnight. The cells were then harvested and cell lysates were applied to dot-blotting and Western blotting analyses (Fig. 4) through the A11 antibody. β-Actin was also detected as a control protein. The A11-reactive protein dots from dot-blotting and the oligomer proteins bands with 14–17 kDa molecular weights from Western blotting were analyzed. We showed that curcumin significantly reduced A11-reactive oligomer levels compared to control in dot-blotting analysis (Fig. 4a,b) and Western blotting analysis (Fig. 4c,d).

## Conclusion

We showed that genetic and pharmacological components led to robust AD-related pathologic protein changes, which can be reliably detected by our nFTP technology in single live mammalian cells. Moreover, we identified significant roles of curcumin in attenuating the changes in intracellular A11-reactive oligomers and Aβ42 proteins caused by LPS and a FAD mutation. Intriguingly, the suppression of oligomerization dominates curcumin’s anti-inflammatory effects. Our present study provided further molecular mechanistic insights for the roles of curcumin and analogs as potential therapeutic small molecules for AD. Specifically, this study provided strong evidence for the roles of curcumin in anti-oligomerization. Notably, intracellular oligomerization of Aβ peptides and protein aggregation are early AD events^23, 37^, which may cause cell death via mechanisms related to endoplasmic reticulum stress, endosomal/lysosomal leakage, caspase-3 activation and mitochondrial dysfunction^43, 44^. Furthermore, our results also showed the well-known effects of curcumin in anti-inflammation^13^.

In summary, as increasing evidence is supporting that intracellular Aβ proteins are critical for AD pathogenesis, nFTP provides a robust and quantitative system to characterize these early pathologic events at a single-cell level. Collectively, the nanoscale intracellular detection system provides a unique and advanced method to evaluate live-cell responses to pharmacological or genetic modulators, thus could become an integral tool for phenotypic drug discovery.

## Materials and Methods

### Cell culture

We used naive Chinese hamster ovary (CHO) cells and previously reported 7PA2 cells stably expressing APP^Indiana^ with high-level Aβ and oligomers^23, 45^. Naive cells were cultured on regular tissue culture plates in Dulbecco’s modified Eagle’s medium (DMEM) supplemented with 10% fetal bovine serum, 2 mM L-glutamine, 100 unit/ml penicillin, 100 μg/ml streptomycin. Medium of 7PA2 cells was supplemented with 200 μg/ml G418.

### Chemicals and antibodies

Curcumin and LPS were from Sigma, and their stock solutions were dissolved in DMSO and PBS, respectively. The A11 antibody was from Millipore (AB9234) and was reactive to oligomeric proteins^18, 36, 42^. The Aβ42 antibody was also previously tested in our group^38^ and was from Abcam (Ab10148). The TNF-α antibody was from Abcam (Ab9739). The β-actin antibody (from Sigma; 1:10,000) were used as house-keeping protein in the immune-blotting analysis. The HRP-conjugated secondary antibodies (anti-mouse and anti-rabbit; 1:10,000) were purchased from Pierce.

### Nanoplasmonic Fiber Tip Probe (nFTP) Fabrication

The nFTP was fabricated by wet-etching an optical fiber (SM28, Thorlabs Inc.) with hydrogen fluoride chemistry by precisely monitoring and controlling the etch time^39^. Gold nanorods were dispersed on the coverslip and then picked up onto the nano-tip of the nFTP using a micromanipulator under the dark field imaging of an inverted microscope. A UV-curing optical adhesive (Norland NOA 128) was used to strengthen the adherence a nanorod to the tip. The nFTP had a tip size of approximately 50 nm. The gold nanorod was 86 nm long and had a 25 nm-diameter cross-section (Nanopartz Inc.).

### Functionalization

One milliliter of cetrimonium bromide (CTAB)-capped gold nanorods (Nanopartz) was mixed with 100 μL of 20 mM solution of 11-mercaptoundecanoic acid (11-MUA, Sigma-Aldrich) prepared in ethanol. The mixture was sonicated for 90 min at 55 °C and kept at room temperature overnight. These nanorods were centrifuged at 5500 rcf for 10 min and redispersed in water to remove the excess 11-MUA. A single nanorod was immobilized on the tip of the nFTP. The assembled nFTP was incubated in 100 mM 1-ethyl-3-(3-(dimethylamino propyl) carbodiimide (EDC, Sigma-Aldrich) with 100 nM of antibodies to the target proteins. To block the nonspecific binding, the nFTP was incubated in the cell culture medium in which fetal bovine serum (FBS) was replaced by 1% bovine serum albumin (BSA).

### Fluorescent imaging

All incubations with curcumin, LPS and control cell samples were prepared by seeding from stock plates and grown for an average of 48 hours. Treated cells were incubated overnight with LPS or LPS/curcumin. Curcumin concentration was 25 μM and LPS was 10 ng/mL. Cell samples were fixed and stained for imaging using the Abcam Immunocytochemistry staining protocol using 4% paraformaldehyde (PFA) in PBS. Both primary and secondary antibodies were 1:1000 dilution with incubation times of 50 mins. DAPI was used to stain the nucleus in water for 10 mins, diluting 1:2000 from 1 mg/mL stock. It was then rinsed 3 times for 5 min with water. Secondary antibodies for immunostaining were goat anti-rabbit IgG Alexa Fluor 488 from Thermo Fisher. Fixed and stained cells were mounted in mowiol4–88, and imaged using a confocal microscope with 63 × oil immersion lens.

### Cell lysis and protein analysis

The methods for cell lysis and protein analysis were previously reported^17, 46^. Briefly, cell lysates were generated by lysing cells in M-PER (Mammalian Protein Extraction Reagent) with 1 × halt protease inhibitor cocktail (Thermo Scientific). The lysates were collected, centrifuged at 10,000 g for 20 minutes. The supernatants were collected and an equal amount of protein was applied to immuno-blotting analysis. For dot-blotting analysis, samples were applied to a nitrocellulose membrane. For Western blot analysis, samples were applied to electrophoresis, followed by transfer to a polyvinylidene difluoride (PVDF) membrane. The blots were then applied to antibody incubation and signal development. β-Actin was used as an internal control. We used the LI-COR Odyssey Fc imaging system to develop the membranes and the software Image Studio from LI-COR was used to demonstrate and analyze the proteins of interest.

### Statistics

P values were obtained from the t-test analysis with one-tailed, two-sample equal variance.
